# Preliminary Assessment of Asymmetric Triangular Riblet Microstructures for Drag Deduction and Fouling Resistance: Numerical Modeling, Fabrication, and Performance Evaluation

**DOI:** 10.3390/mi13122208

**Published:** 2022-12-13

**Authors:** Benjamin W. Hamilton, Remus O. Tutunea-Fatan, Evgueni V. Bordatchev

**Affiliations:** 1 Department of Mechanical and Materials Engineering, Western University, London, ON N6A 6B9, Canada; 2 Automotive and Surface Transportation, National Research Council of Canada, London, ON N6G 4X8, Canada

**Keywords:** bioinspired, drag reduction, fouling resistance, functional surface, precision microfabrication, asymmetric triangular riblets

## Abstract

Several species of plants and animals demonstrate an ability to resist the accumulation of contaminants natural to their environments. To explain this phenomenon, mechanisms that facilitate fouling resistance have to be deciphered. Along these lines, this study is focused on the correlation between drag reduction and fouling resistance for underwater surfaces. This was accomplished by means of a novel microtopography inspired by fish-scales and conceived as a series of asymmetric triangular microgrooves oriented in the spanwise direction. A parametric study involving Large Eddy simulations was carried out to determine the most effective dimensions of the riblets and the results obtained have indicated a 9.1% drag reduction with respect to a flat reference surface. Following this, functional samples were fabricated in acrylic by means of a multi-axis micromachining center and diamond tooling. Surface quality and form accuracy of the fabricated samples were assessed with an optical microscope and optical profilometer. Finally, the fouling resistance of the samples was assessed by subjecting them to a flow of contaminated water. The results demonstrate that a relationship exists between the relative size of the particle and the fouling resistance of the microstructured surface.

## 1. Introduction

Many marine creatures such as mollusks, crabs, sharks, fish, and sea stars demonstrate varying degrees of protection against the accumulation of environment contaminants on their outer surfaces [1,2,3]. This ability is known as fouling resistance and has also been observed in several species of plants. For many of these creatures, this capability is crucial for their survival since they lack the ability to clean themselves. The accumulation of fouling on sharks and fish makes them more susceptible to skin diseases. Fouling also increases the hydrodynamic drag and this results in increased energy needs and/or reduced swimming speeds that lead to susceptibility to predators and food scarcity. Likewise, surface fouling on plants blocks the sunlight required to create the energy required for metabolic processes such as photosynthesis.

Fouling also affects man-made systems. For instance, the underwater surfaces of ship hulls, buoys and offshore structures are susceptible to the accumulation of aquatic organisms (barnacles, algae, etc.). These organic accumulations roughen the surface of ships and this increases drag and thereby stress on the propulsion system, ultimately leading to increased fuel consumption rates. The aquatic growth also increases corrosion rates and thus reduces equipment lifetime. The annual global cost associated with fuel consumption, cleaning and maintenance is estimated to be more than $150 billion USD [4,5]. To combat fouling, ship hulls have been coated with tin-based biocide paints that provide protection for up to seven years. These special paints leach out compounds of tin that poison the nearby organisms and prevent their adhesion. Nonetheless, while being effective at hull fouling prevention, these paints proved to be hazardous to non-targeted marine species and eventually to humans that might be consuming them. By 2008, a global ban was issued on the use of any paint containing specific compounds of tin [6]. While alternative coatings were developed, they were reportedly being less effective while being associated with a certain level of toxicity to aquatic organisms. Because of this, alternative biocide-free fouling resistant solutions became an important avenue to be explored.

Along these lines, much of the work conducted recently was focused on the understanding of the natural defense mechanisms employed by various members of the flora and fauna. So far, researchers have proposed five mechanisms capable to lead to fouling resistance: surface topography, mucous secretion, flexion, sloughing, and surface energy [2]. Each of these mechanisms can be employed on its own or in combination with others. For engineering applications, the most common fouling resistant mechanism is constituted by surface topography, typically because of its passive nature as well as its applicability across a wide range of materials.

To date, sharkskin is typically regarded as one of the best models of fouling resistant topography. An adhesive film mimicking its microtopography is presently available on the market [7,8]. The skin of sharks is comprised of many tooth-like denticles each comprised of several microriblets that are positioned parallel with the direction of the flow. While the basic geometry of the microstructures does not vary much among shark species, the length of their scales is variable and could be anywhere between 30 µm and 300 µm [9,10]. Past research efforts have shown that bio-inspired shark skin topography can reduce the friction drag associated with turbulence by nearly 10%. Subsequent studies found that shark skin-inspired microriblets are capable of reducing surface fouling by as much as 86% [11]. It is believed that a complementary correlation exists between the fouling resistance of this surface and its ability to reduce drag, possibly because the rapidly moving water layer located in the close proximity of the structured surface carries away particles and organisms that would otherwise settle and adhere to the surface. Nevertheless, the bioinspired shark skin topography does not seem a decisive solution to surface fouling. In a long-term field trial of shark skin-inspired topography, the fouling deterrent effect was significantly diminished after six months [12]. This loss in performance prompts the need for further development, particularly with respect to microstructure geometries that are inspired by other types of native fouling resistant surfaces. Additionally, investigating the correlation between drag reduction and fouling resistance for alternative surfaces may reveal fundamental underlying mechanisms employed by nature.

Along these lines, fish scales have also been associated with drag reduction in low Reynolds flow [13]. In this case, the proposed mechanism was a delayed onset of the turbulent flow and the increased drag associated with it. In this regard, Muthuramalingam et al. [13] demonstrated a 3% drag reduction for fish scales with respect to a flat witness surface. Although fish scales were not investigated to the same extent as shark skin structures, the preliminary studies of fish scales showed promising fouling resistant properties [14]. The hydrophobicity/oleophobicity demonstrated by biological fish scales is believed to contribute to their fouling resistance. In this context, it becomes increasingly clear that a better understanding of the correlation between surface microstructures and drag reduction is quintessential for generation of “man-made” surfaces whose fouling resistance would equal or exceed that of their natural counterparts.

Building on this, the primary objective of this study was to examine the drag reducing mechanisms associated with the bioinspired ribletted microstructures along with their ability to resist fouling. For this purpose, a novel riblet geometry was analyzed as a possible candidate of a drag reduction surface topography therefore allowing for the investigation of the anticipated correlation leading to fouling resistance. Computational fluid dynamics (CFD) models of the topography were performed for a range of microriblet dimensions in an attempt to evaluate drag and establish functional geometries. Samples were then microfabricated in PMMA (polymethyl methacrylate) followed by the analysis of form accuracy and surface quality for surface microstructures. Finally, the fouling resistance of the samples was investigated by means of a gravity driven channel and contaminated flow.

## 2. Parametric Design and Numerical Simulation

The triangular riblets developed for this study represent one of the possible geometric embodiments of microgrooves. These structures fit well within the V-groove family of microfeatures that are typically used in surface functionalization. Possible applications of V-grooves include microfluidic systems [15], friction control [16], and micro-optics [17]. Nonetheless, the geometry presented in this work is inspired from fish scales in an attempt to be fouling resistant as well as characterized by drag reduction capabilities.

### 2.1. Parametric Design of Asymmetric Triangular Riblets

Along with drag reduction and fouling resistance, fish rely on their scaled surfaces for maneuverability, camouflage, and protection. Although biologists have identified several types of fish scales, the cycloid scale tends to be the most prevalent. While the size and shape of fish scales have a number of species-dependent particularities, certain commonalities are inherently present for all of them [18].

**Figure 1:**
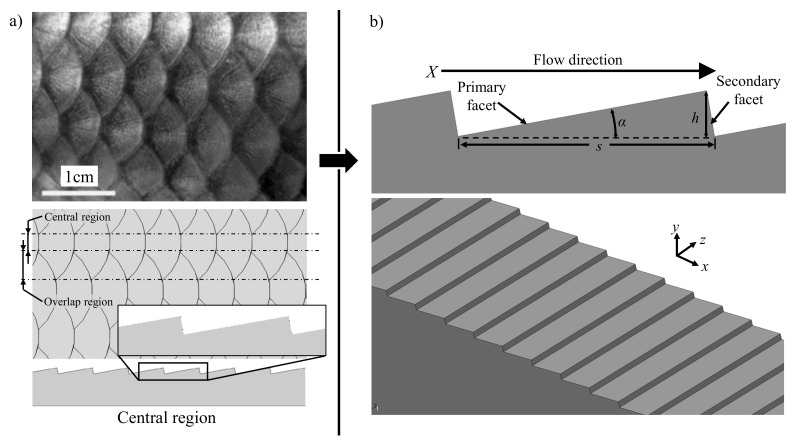
Overview of fish scale geometry: (**a**) topography of typical fish-scales (adapted from [16]), and (**b**) proposed design of asymmetric triangular riblets.

The taxonomy of cycloid fish scales derives its name from the semicircular shape of individual scales (Figure 1a). Additionally, Liu et al. [14] illustrated the hierarchical nature of fish-scales through images captured with a scanning electron microscope (SEM). On the surface of each scale are papillae that are 100–300 µm long and 30–40 µm wide. Furthermore, nanometric-size structures exist on the surface of each papilla. This complex hierarchy is believed to contribute to their superoleophobic property. The scales are arranged in an overlapping pattern along the entire fish. The anteroposterior arrangement of the scales resembles that of asphalt shingles used in residential roofing in an attempt to adequately drive the water flow. As mentioned above, biological samples of this topography have demonstrated drag reduction up to 3% in low Reynolds flow.

To investigate the contributing effects of a complex and hierarchical 3D topography, asymmetric triangular riblets (ATR) have been introduced as a simplified fish scale-inspired geometry. As depicted in Figure 1a, biological fish scales have two distinct regions: the central region and the overlap region. In the overlap region, scale edges meet at alternating angles, while the protruding edges of the central region are perpendicular to the direction of flow. Hydrodynamic effects of the central region are expected to be less complex compared to the overlap region. As such, the proposed ATR structures represent the characteristics of the central region. Therefore, the two-dimensional simplification is a repeating pattern of scalene right triangular riblets, oriented perpendicular to the nominal flow direction and whose 90° corner forms the trough of the groove (Figure 1b). The two-dimensional nature of the topography describes its constant cross-section whereas facet nomenclature is indicative of its exposure to the flow. In reference to the peak of each groove, the primary facet is upwind of the flow direction, while the secondary facet is downwind. The angle of the primary facet (*α*), measured with respect to the horizontal direction and the riblet period or spacing (*s*) are sufficient to fully define the ribletted geometry. The groove height (*h*) is a dependent variable measured as the distance between the two horizontal planes formed by the peaks and troughs of the grooves. Groove height can be calculated as:

(1) $$h=\frac{s}{2}\sin2\alpha$$

A limited number of studies on the drag reduction potential of transverse riblets have been conducted in the past and they generally yielded conflicting results. More specifically, Gruneberger et al. [19] have experimentally investigated trapezoidal riblets across a range of scales and found no drag reduction effect. Instead, their results closely match the theory for equivalent sand roughness drag. Conversely, Li et al. [20] have demonstrated drag reduction for transverse rectangular riblets in both numerical simulations and water channel experiments and concluded that these effects are the consequence of a ‘vortex drag reduction effect’ according to which the recirculating fluid trapped in the riblet valleys causes a net reduction in drag.

### 2.2. Numerical Simulation of Drag Performance

In the absence of established flow characteristics of ATR, a parametric study was carried out for a range of dimensions of *α* and *s* [21]. The baseline geometry had *α* = 10° and *s* = 585 µm thus resulting in a 100 µm height. Although the effective feature height is largely dependent on the flow conditions to which it is exposed to, previous studies on riblets aligned with flow direction have demonstrated that the effective dimensions are somewhere between 50 and 500 µm for conditions similar to those tested in the present study. Furthermore, the laminar subregion of the boundary layer, located between the surface and approximately 200 µm normal to and above the surface, was considered an upper bound for feature height. Therefore, the range of dimensions chosen in the previous study [21] results in *h* between 20 and 150 µm. Variables *α* and *s* were independently changed to capture their individual effects.

Numerical simulations were conducted by means of Ansys^®^ Fluent 2019 and the large eddy simulation model (LES), along with the Smagorinsky-Lilly subgrid model [22]. This approach has proven effective in similar studies evaluating the drag reduction potential of various surface topographies [23]. In contrast with the direct numerical simulation (DNS), LES represents a more computationally efficient method because it applies a mathematical model to the smallest length scales, thus decreasing the mesh density required to directly resolve them.

**Figure 2:**
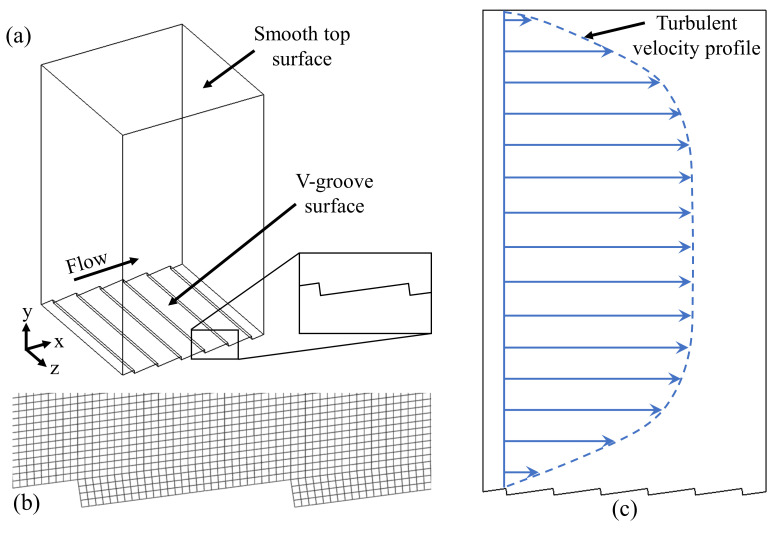
Methodology of numerical simulation of drag performance: (**a**) computational domain for 8° case, (**b**) mesh detail near ATR topography, and (**c**) anticipated mean velocity profile through domain.

The computational domain consists of a channel modeled with the ATR geometry on the bottom wall and a flat surface on the top. A no-slip boundary condition was applied to these surfaces. Therefore, the flat surface could be used as a reference to calculate the relative drag change of the ATR topography in accordance with the proposed methodology of the computational domain presented in Figure 2. The *X*, *Y*, and *Z* axes of the computational domain denote the streamwise, wall-normal, and spanwise directions, respectively. Periodic boundary conditions were applied to the inlet and outlet, as well as to the left and right surfaces. These boundary conditions are used to approximate the flow in a large domain while considering a small region of that domain. The paired boundaries behave in such a manner that allows flow exiting one side to be immediately introduced on the corresponding/opposite boundary. Thus, coherent turbulent structures *pass through* rather than *interact* with the four vertical boundaries.

Because the functional drag reduction mechanism of fish scales leads to a delayed turbulence onset, the Reynolds number for these simulations (*Re*) is of utmost importance:

(2) $$Re=\frac{U_{l}L}{v},$$

where *U_l_* is the centreline velocity, *v* is the kinematic viscosity of the fluid, and *L* is the characteristic length scale, i.e., channel half height. The transition to turbulence is generally considered to occur at 2000 < *Re* < 4000. Therefore, the most pronounced drag reduction effect is expected to occur within this transition region. The final channel height was 6 mm, while the dependence on the Reynolds number was investigated with two velocities, 0.75 m/s and 1.0 m/s, corresponding to *Re* = 2250 and *Re* = 3000, respectively. The streamwise and spanwise dimensions of the domain were both 3.5 mm.

In order to accurately calculate the flow field near the top and bottom surfaces, the discretization scheme reduced cell sizes near these surfaces to 20 µm in all three cartesian coordinates. Cell sizes were increased toward the domain centerline where the calculation of the flow field is not pertinent. This method resulted in a computational domain with 170, 84, and 170 tetrahedral cells in the *X*, *Y*, and *Z* directions, respectively. This grid resolution compares well with that used in the LES study of Martin et al. [23], and resulted in a computational domain comprised of approximately 2.5 million cells. The timestep size (Δ*t*) was calculated to maintain a Courant number (*C*) of one:

(3) $$\Delta t=\frac{C\Delta x}{U_{l}},$$

where Δ*x* is the streamwise cell length such that Δ*t* ≈ 2 × 10^−5^ s. This value favors solution stability by ensuring that fluid parcels do not travel more than one cell between each timestep.

The change in drag (Δ*D*) is typically calculated with reference to a flat surface in identical flow conditions:

(4) $$\Delta D\frac{\left(D_{s}-D_{f}\right)}{D_{f}},$$

where *D_s_* is the drag force on the structured surface and *D_f_* is the drag on the flat reference surface. According to this definition, negative values correspond to drag reduction, and positive values to drag increases with respect to the reference flat surface. In the case of perpendicular riblets, the total drag is a combination of friction drag—caused by the shear force—and pressure drag. The pressure drag component is a consequence of the flow separation and recirculation as it passes over the riblet tip and into the valleys across the secondary facet.

**Figure 3:**
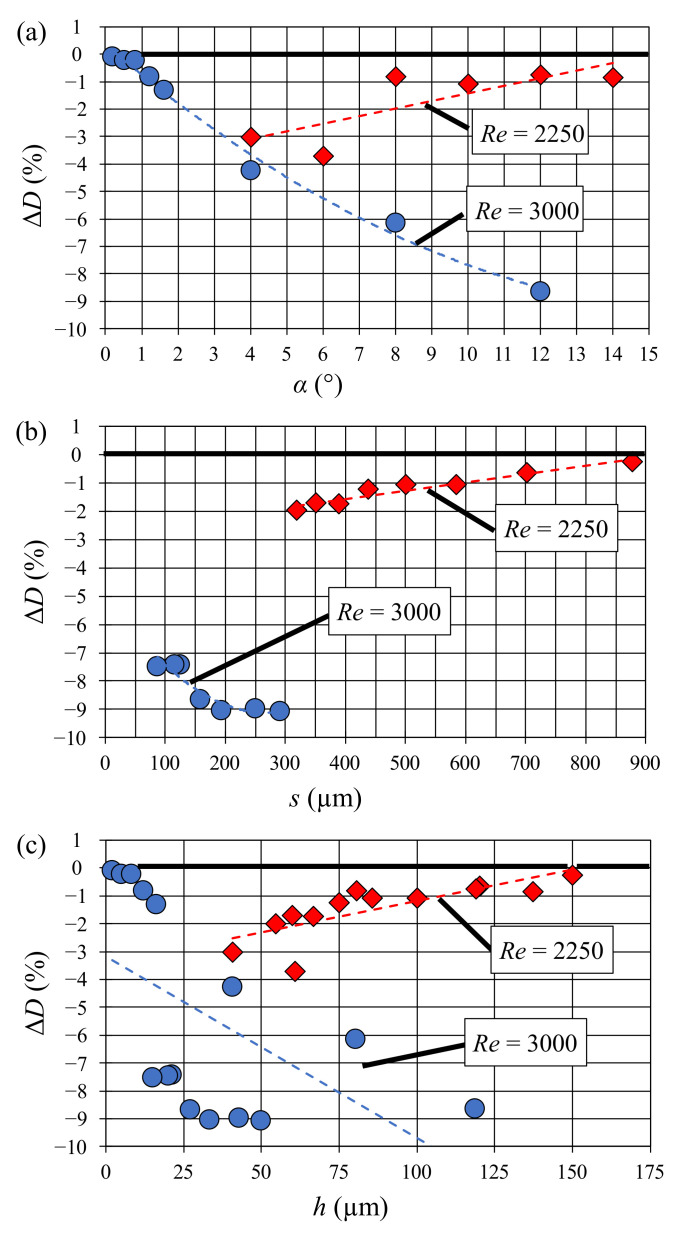
Simulation results: dependence of drag reduction on the principal parameters of the riblet geometry: (**a**) angle of primary facet (**b**) spacing between riblets, and (**c**) riblet height.

Transient simulations were run while monitoring the drag reduction performance in order to determine when the simulation had reached steady state. This process took an average of 15 s of real time or 2500 CPU hours. Steady state was calculated by comparing sequential time averaged values of the change in drag ($\bar{\Delta D}$) as it converged to a single value. Simulations were stopped when the difference between iterations was less than 0.01%. Drag reduction was demonstrated in all cases, with a maximum reduction for large angles at *Re* = 3000 (Figure 3). The drag reduction performance increased as feature height decreases for *Re* = 2250, while the opposite appeared to be true for *Re* = 3000. Irrespective of this, ATR topography proved to be able to reduce the fluid drag for Reynolds numbers within the transition region possibly due to the vortex drag reduction effect described by Li et al. [20]. Overall, this topography seems to be suitable to investigate the correlation between fouling resistance and drag reduction.

## 3. High Quality Precision Microfabrication of Functional ATR Samples

While fouling could be regarded and/or modeled as a multiphase flow with suspended particles, the fouling mechanisms responsible for settling and adhesion to the surface are still not well understood. As such, empirical studies were preferentially conducted in the past [11,24,25]. To determine the functional performance of the asymmetric triangular riblets (ATRs) with respect to their fouling resistance, the present study aims to expose several ATR-structured surfaces to a flow of contaminated water followed by the quantification of the resulting particle settlement.

When it comes to techniques used for fabrication of large scale arrays, several approaches have been employed in the past: machining, additive manufacturing, and resin casting [26]. Along these lines, Gruneberger et al. [19] demonstrated a fouling resistant process involving a microstructured paint layer. This highly technical process—developed primarily for large surfaces of planes and ships—offers a relatively limited flexibility with respect to the geometry of the structured surface. Furthermore, the mechanical properties of the fouling resistant surface are limited to those of the polyurethane resin forming the base of the coating.

In recent years, additive manufacturing processes have become more attractive. Along these lines, Li et al. [20] manufactured several 200 × 100 mm arrays of rectangular riblets by means of the fused deposition modeling (FDM) technique. The process demonstrated in their study was able to achieve an accuracy of 100 µm that is, however, inadequate for the riblet dimensions considered in this study. Moreover, the inherent performance of the additive manufacturing process yields a specific hierarchical surface roughness that introduces undesirable downstream effects on the intrinsic nature of the flow.

Precision microfabrication of functional riblets by single point cutting and conventional micro-milling technologies have been shown to achieve high form accuracy and optical surface quality [27,28,29,30]. Further to these benefits, the widespread use and technological maturity of the micromilling performed with rotational tools offer a degree of simplicity and flexibility that is simply not available with other fabrication techniques.

### 3.1. High-Precision Multi-Axis Micromachining System

**Figure 4:**
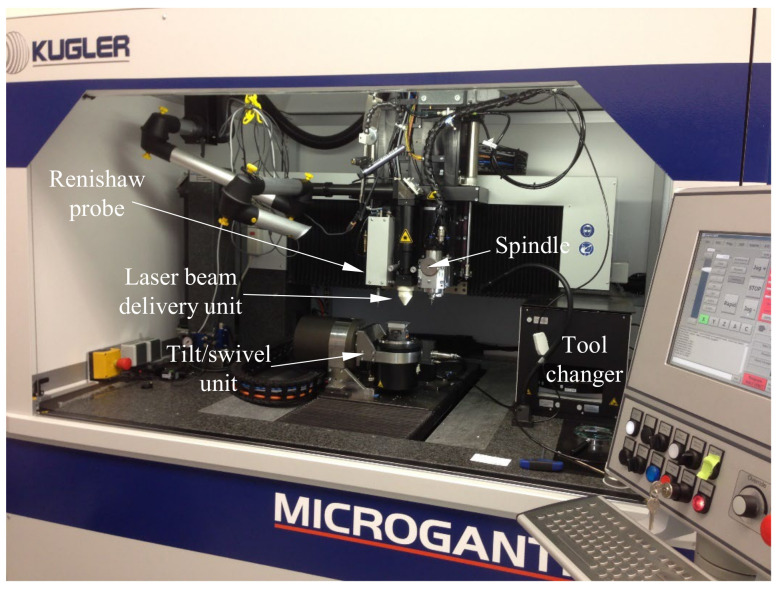
High precision micromachining system.

In this study, sample fabrication was performed on a modular, multi-functional Kugler nano5x micromachining system (Kugler GmbH) equipped with diamond micro-milling tools (Figure 4). The system integrates several micromachining technologies along with the measurement instrumentation and it was used in the past to fabricate functional part prototypes and tooling (inserts, molds, dies, stamps, electrodes, etc.) with optical surface quality in excess of *R_a_* < 10 nm, and high aspect ratios [31,32]. Various technologies including single point diamond cutting and laser-based irradiation can be used for this purpose, the aforementioned Kugler system being capable to integrate a variety of tools.

The system performs workpiece alignment and post-machining geometry verification by means of a Renishaw touch probe characterized by a measurement accuracy of ±500 nm. Online tool measurements (length and diameter) were performed by means of an integrated Blume laser tool setting sensor that has a measurement repeatability (2σ) of 100 nm. The micromachining system includes three linear axes (*X*, *Y*, and *Z*) and two rotary axes (*A* and *C*). The motion stages are suspended on air bearings and have a positional resolution of 10 nm with a static positioning accuracy of ±100 nm, dynamic positioning accuracy of ±1.0 µm, and has demonstrated a cutting accuracy of ±1.0 µm. All three linear axes have a straightness within ±800 nm per 100 mm of travel.

### 3.2. Microfabrication of ATR Samples by 3+1-Axis Micromilling

**Figure 5:**
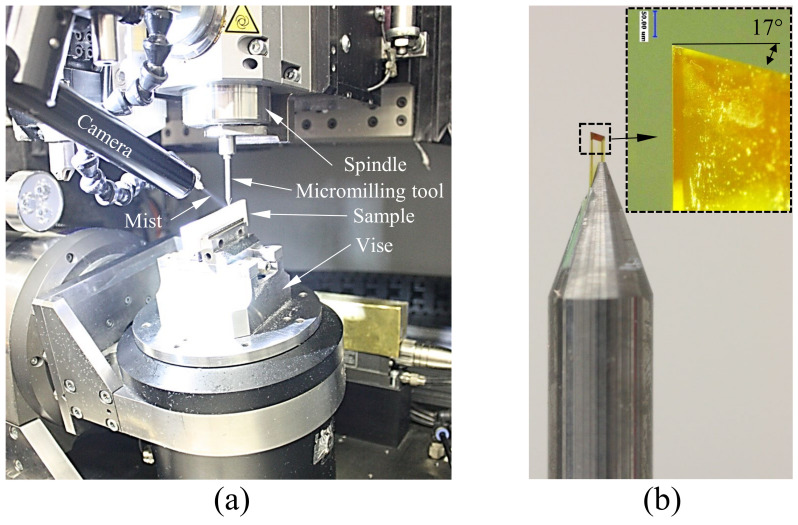
ATR fabrication: (**a**) four-axis micromilling setup, (**b**) cutting tool geometry.

Functional ATR samples were fabricated in acrylic by means of the four-axis micromachining setup depicted in Figure 5. Cutting was performed with a 1.0 mm diameter monocrystalline diamond end mill. The 17° bottom clearance angle was added to reduce the amount of contact between the tool and the machined/finished surface. Nonetheless, this large relief angle also provides an additional clearance for the removal of the chips. The vertical cutting edge of the tool was aligned with the principal axis of rotation for the tool, thus enabling generation of the desired 90° angle between the primary and secondary facets of the ATR geometry.

Initially, a rectangular workpiece of 75 × 20 × 3 mm was fixed in a vise mounted on the tilt-swivel unit. The 20 mm dimension was set parallel to the *Z*-axis of the machine tool. The workpiece was aligned with the *Y*-axis by rotating the *C*-axis to ensure an *X*-axis deviation within 1 µm. This was required to maintain the perpendicularity of the ATR topography to the workpiece long side. The top surface (75 × 3 mm) was then tilted around the *A*-axis in order to align it with the *XY*-plane. This alignment was followed by a facing operation to ensure the flatness of the initial surface was within the desired 1 µm target. While not a critical operation for precision microfabrication, this particular operation prepared the top surface for further precision micromilling of the ATRs.

In the next step, the center of the micromilling tool was aligned to the top left corner (negative *X* and positive *Y*) of the horizontally located sample. This alignment of the micromilling tool with respect to the flat workpiece enabled the 3+1-axis micromachining that takes place when the workpiece is indexed by a 10° rotation of the *A*-axis. Finally, the micromachining of the ATRs was performed in accordance with a preprogrammed three-axis tool path trajectory.

The rotational velocity of the tool was set at 42,000 rpm, a value that corresponds to a cutting speed of 2.2 m/s. This value was proved to be adequate for the achievement of the optical surface quality [33]. Cutting operations involved a chip load of 10 µm/tooth (or 420 mm/min), that was also chosen to prioritize surface finish over the rate of material removal. The machine kinematics developed for this geometry allowed single-axis cutting motions. The *A*-axis index aligned the primary facet parallel with the *XY* plane of the machine coordinate system (MCS), and as a consequence, the secondary facet was oriented parallel to the *XZ* plane. It is also necessary to mention that the indexing motions around *A*-axis shifts the location of the micromilling tool with respect to the workpiece location and therefore additional *XYZ* shifts of the micromilling tool were required to cut grooves and fabricate ATRs.

**Figure 6:**
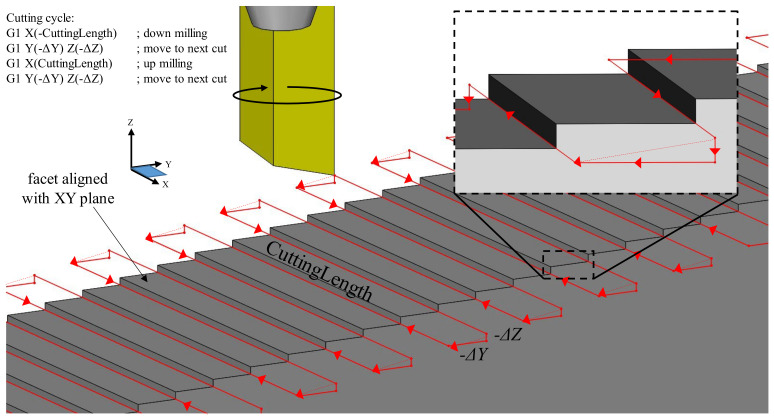
Cutting strategy employed during four-axis micromilling of ATR samples.

Each groove was then cut in a “zig-zag” approach that alternated between climb and conventional milling on consecutive grooves (Figure 6). Conventional machining knowledge suggests that climb (down) milling produces a superior surface finish with respect to its conventional (up) counterpart [34]. Nonetheless, this generally applies to rather ductile metals such as steel and aluminum. By contrast, the authors of a recent study [35] found that conventional milling produced (on average) a superior surface finish during micromilling of PMMA, noting that cutting temperatures above the material’s glass transition temperature had a detrimental effect on surface quality. Therefore, a cutting strategy strictly employing conventional milling might be able to improve the overall surface finish of the fabricated array. The additional positioning motions required for this type of strategy will increase the tool path length by a factor of approximately two. Thus, the proposed fabrication approach represents a trade-off between manufacturing time and resulting surface finish. To mitigate the damage caused by high cutting temperatures, pressurized coolant mist was used throughout the cutting process.

As shown by the red lines in Figure 6, the tool overshoots the *X*-axis width of the ATR with a distance that is at least equal to that of the tool radius. This ancillary motion ensures the tool clears the workpiece while the subsequent positioning motions align the tool with the next groove to be cut. Therefore, the cutting path length for each groove is:

(5) ΔX=W+d,

where *W* is the width of the workpiece while *d* represents tool diameter.

When referring to machine coordinate system (MCS), positioning motions involve both *Z* and *Y* axes and are calculated as respective functions of *s* and *α*:

(6) ΔY=scosα,

(7) ΔZ=ssinα,

The tool position depicted in Figure 6, corresponds to the end of the down milling cut (−*X* direction) in the included four lines of G-code. The subsequent tool motion (move to next cut) is a vector addition of Δ*Z* and Δ*Y* and aligns the tool with the next groove to be cut. Line three of the G-code is a positive *X* motion and cuts the groove in an up milling motion. Finally, the tool is repositioned in line four with a positioning move identical to the one used in line two. This four-line algorithm was repeated along the length of the workpiece until all grooves were generated.

For an array cut in a workpiece of *x* by *y* dimensions having the grooves aligned with *X*-axis, the minimum tool path length (*L*) would simply be the summation of cutting and positioning motions for each groove:

(8) $$L=\frac{y}{s}(\Delta X+\Delta Y+\Delta Z)$$

A strategy implementing climb milling alone would require additional positioning motions, thus effectively increasing the Δ*X* term by a factor of two.

To illustrate the validity of the proposed approach, three samples were fabricated with a two-fold purpose: a comprehensive analysis of the fabricated geometry followed by a fouling resistance study. As outlined in the results of the numerical simulation, drag reduction was present for the full range of angles and spacing considered. Therefore, according to the hypothesized correlation between drag reduction and fouling resistance, the dimensions of the ATR topography within the range used in simulations are expected to mitigate fouling buildup.

The height of the geometry was selected based on its relative size in comparison to the 16 µm characteristic dimension of the particles used in the fouling study and their anticipated interaction with the topography. The three heights selected were 8, 16, and 32 µm, corresponding to a ratio of groove depths to particle size of approximately 0.5, 1.1 and 2.1, respectively. For *α* = 10° the selected spacings were 46.8, 93.6, and 187.1 µm. While these dimensions are generally smaller than the biological fish scales depicted in Figure 1, they fall within the dimensions investigated in the numerical simulation and therefore will provide a drag reduction effect thus allowing for the investigation of its correlation with fouling resistance.

Arrays were fabricated on the narrow edge of 2.9 × 75 × 20 mm PMMA (polymethyl methacrylate) samples. This polymer was chosen for its wide use in prototyping functional surfaces. Previous studies have shown that PMMA is a good material for producing micro/nano structures with a high-level of form accuracy and surface quality [28,36]. Additionally, the low surface energy of PMMA reduces the surface chemistry between the microstructures and silicon carbide particles to be used in the upcoming fouling investigation.

By applying Equation (7), the tool path length for the 8, 16, and 32 µm heights yield as 6.34, 3.21, and 1.65 m. For each of these, the cutting proportion of the tool path represents more than 73% of the total length. Evidently, the largest features result in the shortest tool path. This is a consequence of the toolpath length dependence on the number of riblets located along the 75 mm dimension of the workpiece.

### 3.3. Microfabrication by 4-Axis Micro-Milling Using Rotating Tool Center Point (RTCP) Method

The tool motions described above represent incremental distances from one groove to the next in MCS. These calculations assume that the tool has been accurately aligned with the workpiece such that grooves are being cut and formed as expected. The alignment procedure introduces the concept of a work offset defined as a vector that captures the location of the work coordinate system (WCS) with respect to the MCS.

**Figure 7:**
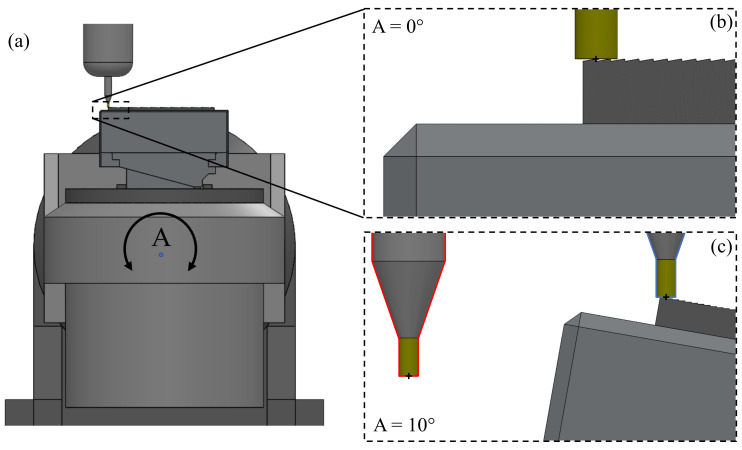
Four-axis micromilling of ATR samples: (**a**) machine tool overview, (**b**) tool orientation for A = 0°, and (**c**) tool orientation without RTCP on as 3+1-axis machining (red outline) and with RTCP on (blue outline).

For three-axis machine tools, work offset is a constant vector determined during the machining setup procedure. For machine tools with rotational axes, the workpiece moves relative to the tool as the orientation of the rotational axes changes. For example, an *A*-axis rotation of 10° is depicted in Figure 7. The tool center point is initially located at the tip of first riblet (Figure 7b). As the part rotates about the center of rotation for *A*-axis, the resulting tool misalignment is shown in Figure 7c (outlined in red). In this case, tool center point is no longer located at the tip of the first riblet, and therefore it must be realigned. For this purpose, the programmer must rely on machine kinematics in order to reposition the tool over the riblet tip.

**Figure 8:**
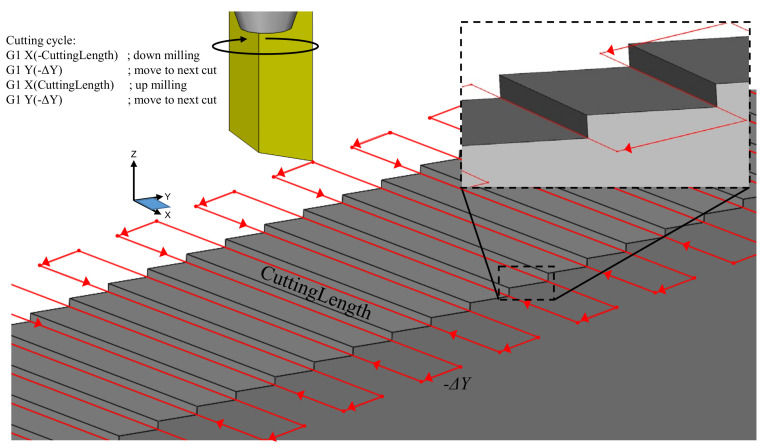
Tool path trajectory for four-axis micromilling involving RTCP.

Alternatively, a built-in function called rotating tool center point (RTCP) can be used. As the name implies, RTCP effectively relocates the center of rotation to the tool tip through a transformation function. When RTCP is applied, rotational movements do not change the location of the tool with respect to the workpiece but tool tip follows the workpiece as it rotates, as depicted in Figure 7c (outlined in blue). Because of this, NC code does not need to account for machine kinematics thus leading to simplified calculations and code portability to other machine tool kinematic configurations. The calculation advantage of RTCP is outlined in Figure 8. As shown here, the positioning motions described in lines two and four of the G-code have a single −Δ*Y* motion, rather than the −Δ*Y* and −Δ*Z* motions that are required when RTCP is not invoked. Furthermore, the tool motions are respective to the WCS attached to the workpiece. As such, Δ*Y* solely depends on the periodic spacing:

(9) $$\Delta Y_{\mathrm{RTCP}}=s$$

An added benefit of calculated tool motions not involving *α* is that the program can be quickly adapted for other ATR geometries with the same spacing but different inclination angles. This can be achieved by just changing the line of code pertaining to the *A*-axis indexing motion. The cutting motions of lines one and three remain unchanged when RTCP is active.

A further benefit of RTCP is related to its ability to account for the geometric errors of the machine tool. Theoretically, the three linear axes of a machine tool are mutually orthogonal to each other and each rotational axis is parallel with its respective linear axis. However, geometric errors are inherently present due to manufacturing inaccuracies alongside with any damage incurred over the lifespan of the machine tool [37]. These errors directly affect the relative position of the tool with respect to the workpiece and eventually lead to workpiece dimensional errors. These errors can be reduced through routine maintenance and alignment procedures. However, they cannot be eliminated and hence they must be factored in during precision micromachining operations. One option available for this purpose involves the use of RTCP that dynamically accounts for the absolute location of the axes of rotation as well as their true directions.

### 3.4. Form Accuracy and Surface Quality of Micromilled ATR Samples

**Figure 9:**
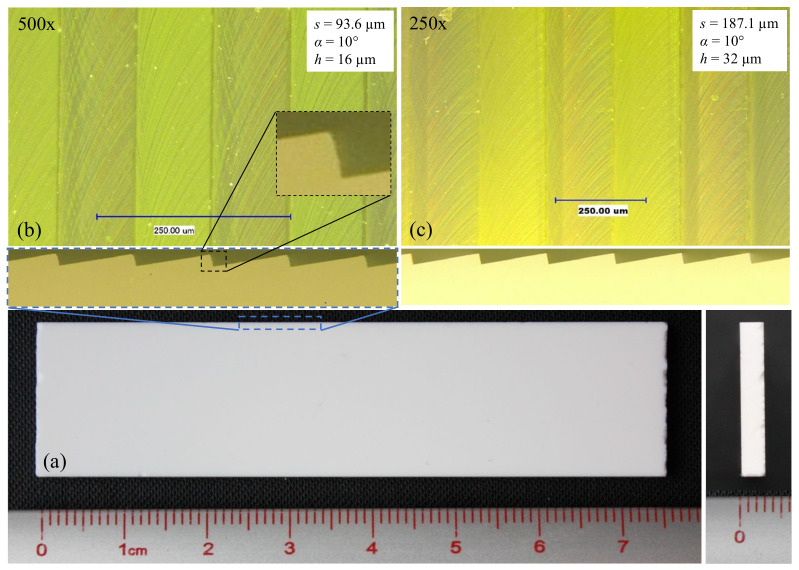
Example of fabricated ATR samples: (**a**) overview, (**b**) surface detail for *h* = 16 µm, and (**c**) surface detail for *h* = 32 µm.

The form accuracy and surface topography of the fabricated arrays were analyzed with an optical microscope with attached CCD camera, and a white light 3D optical profilometer. The representative images of the machined topography shown in Figure 9 demonstrate the high form accuracy achieved with this fabrication strategy. The side views suggest the trough of each groove is formed by a sharp 90° angle, while each peak displays a limited amount of chipping.

**Figure 10:**
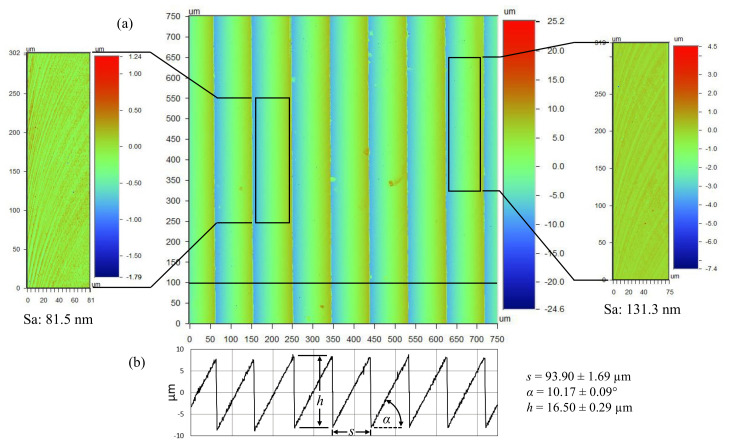
Surface topography of *h* = 16 µm ATR sample: (**a**) areal surface roughness of the conventionally (**left**) and climb (**right**) milled primary facets and (**b**) ATR form geometry.

The changes in color of the primary facets suggest a difference in surface roughness between climb and conventional milling. The extent of this difference was quantified by means of an optical profilometer (Figure 10). The surface roughness on each facet of the ATR microstructures introduces a surface hierarchy that could potentially influence the overall fouling resistance performance of each sample. Liu et al. [14] attributed surface hierarchy to hydro/oleophobicity leading to fouling resistance. Additionally, an investigation of nanotopographies on stainless steel surfaces found that nanoscale surface roughness prevented the adhesion of bacteria compared to an electropolished surface [38]. Therefore, surface roughness has been shown to influence fouling resistance. Since the present investigation is on the fouling resistance of ATR structures, decreasing differences between the surface roughness of each sample was achieved by means of similar cutting parameters used throughout the fabrication process.

The areal surface roughness (*S_a_*) was measured at several locations of the primary facets in order to determine the average roughness of climb and conventional milling. For the sample with 16 µm tall structures, the conventional milled facets had a mean roughness of 81.5 nm, while the mean roughness for the climb milled facets was 131.3 nm. A similar difference was noticed for the sample with 32 µm structures: 101.7 nm and 142.1 nm, respectively. Finally, the 8 µm sample showed no visible difference between climb and conventional milling facets and was characterized by an areal surface roughness of 120.5 nm. Although the micromilling-based strategy failed to generate optical surface quality, the relative difference between each sample is expected to contribute very little to overall fouling resistance performance. Additionally, the characteristic dimensions of the achieved surface roughness is approximately three orders of magnitude smaller than the micromachined structures. As such, the surface roughness is not expected to interfere with the flow mechanisms responsible for drag reduction and fouling resistance.

Data captured with the optical profilometer was also used to assess the geometric accuracy of the samples. Along these lines, Figure 10b is a 2D plot representing data captured along the horizontal line at 100 µm from the lower edge. These data were used to determine the parameters *h*, *s*, and *α* that were achieved on the fabricated samples. For the 16 µm sample, the average feature height and spacing was 16.5 ± 0.29 µm and 93.9 ± 1.69 µm, respectively. The surface scanning resolution of the optical profilometer was 384 nm along *X* and *Y* axes, and 0.1 nm along *Z* axis. The discrepancy between the intended spacing (93.6 µm) and the one that was practically achieved (93.9 ± 1.69 µm) was regarded as negligible. On the other hand, the 0.5 µm difference in height was larger and it is believed to be a consequence of the angular positioning inaccuracies (10.17° rather than 10°). The 8 µm sample had the lowest absolute error whereas the 32 µm sample had the largest absolute error. This suggests that machining errors tend to increase with the size of the fabricated features.

## 4. Performance Evaluation of Fouling Resistance

To quantify the fouling resistant capability of the ATR topography, the fabricated samples were exposed to a flow of water contaminated with silicon carbide (SiC) particles. Image analysis was used to determine the particle coverage as a percentage of the total surface area. Bixler et al. [24] advocated for the use of SiC particles as an “artificial” contaminant because of their known similarity—in terms of size, shape, and hydrophilicity—with natural dirt. The particles used in this study had an average size of 15 µm and were mixed in water at a mass concentration of 0.50% SiC.

**Figure 11:**
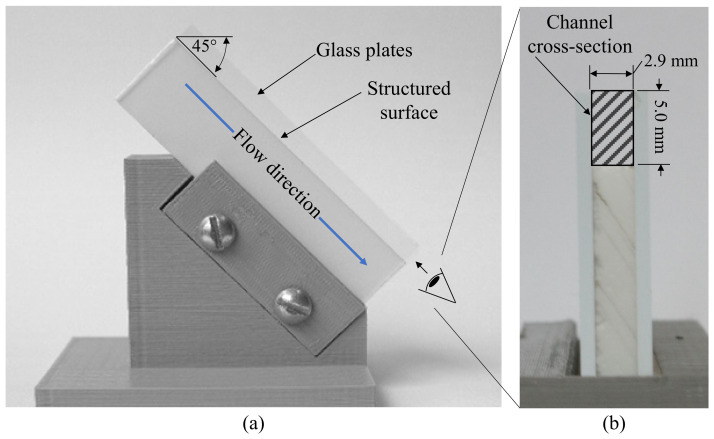
Experimental apparatus (**a**) showing sample inclination angle of 45° and (**b**) view from outlet showing channel cross-section delineated by two glass plates and the structured surface.

A syringe drive was used to direct the contaminated water over the samples at a rate of 1.0 mL/s and for 100 s. The samples were inclined at 45° to facilitate gravity-driven flow over microstructures (Figure 11). In order to control the flow of water over the small 3.0 mm surface of the fabricated topography, a channel was created by placing the sample between two glass plates. In this setup, the plates formed the channel walls, while the ATR topography formed the bottom surface of the channel. The specified flow rate and channel dimensions resulted in a maximum velocity of approximately 0.45 m/s as verified by a high-speed camera. Following the exposure to the contaminated water, samples were removed from the channel and air dried at room temperature for at least 30 min to facilitate the evaporation of the water. This procedure was repeated for each of the three samples.

**Figure 12:**
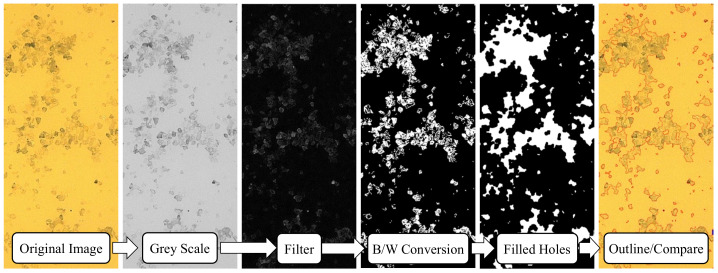
Flowchart of the fouling analysis algorithm (images were truncated for clarity).

A total of eight 1000 × 750 µm images were captured for each sample by means of optical microscope and CCD camera. The images were then analyzed with a Matlab code conceived to quantify the particle-covered area. The main image processing phases are shown in Figure 12. More specifically, after certain image format changing and filtering steps required to enhance the boundary detection, small-sized open “holes”—essentially corresponding to SiC particles—were filled through a multipass process. By contrast, “holes” that were detected to be larger than the dimensions of a single SiC particle were left unfilled and regarded as imaging artifacts. Following this processing, the final binary image (“Filled Holes” in Figure 12) was used to calculate the area fraction of total fouling coverage (FC) as:

(10) $$\mathrm{FC}=\frac{N_{w}}{N_{w}+N_{b}},$$

where *N* represents the number of pixels in the binary image and the subscripts *b* and *w* denote the black and white pixels. The final verification step was used to visually assess the accuracy of the algorithm. For this purpose, black-white boundaries of the binary image were extracted and overlaid as red contours on the original image. In case of obvious mismatches with respect to the original image, algorithm-specific parameters were modified until a superior contour matching was obtained. It is important to note that the preliminary quantifications performed without “hole filling” were erroneous due to the inconsistencies in particle color that led to inaccurate particle/no-particle assessments.

The analysis was repeated for each of the eight representative images captured for each ATR sample (to a total of twenty-four images for the three fabricated samples). For each of ATR samples, the mean fouling coverage was reported after removing the highest and the lowest values of each eight-image dataset. The anticipated interaction between the ~15 µm SiC particles and the fabricated grooves suggests that the 8 µm sample will demonstrate a distinct advantage over the larger 16 and 32 µm microstructures. More specifically, since the particles had almost twice the height of the grooves, it is reasonable to expect that they will be unable to settle into the grooves because they will be washed away by the water. Nonetheless, the experimental results proved to be contrary to this hypothesis since the 32 µm sample demonstrated the least amount of fouling coverage (0.99%), whereas the 8 and 16 µm samples exhibited 2.47% and 2.71% fouling, respectively.

**Figure 13:**
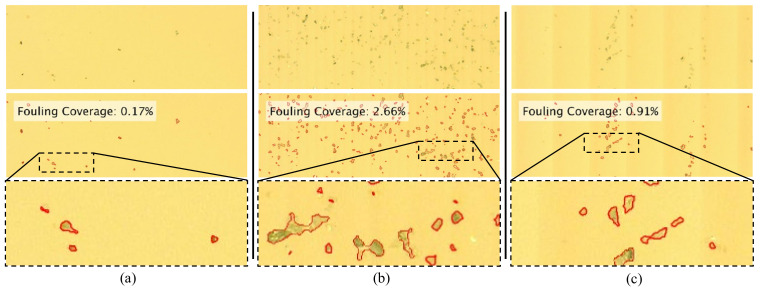
Representative experimental examples of fouling: (**a**) smooth reference, (**b**) 8 µm, and (**c**) 32 µm samples.

To provide a qualitative representation of the fouling phenomenon, the cropped images in Figure 13 illustrate the fouling coverage of a single image from the dataset of eight collected for each of the three ATR samples. The original image is at the top of each column, while the middle image displays the overlaid coverage determined through analysis. The absolute difference between the four samples (including the smooth control) suggests that their fouling performance might not be very different. However, increased exposure time and varying conditions will have to be considered in future studies.

As mentioned above, simulations demonstrating drag reduction were conducted with Reynolds numbers within the transition to turbulence regime. By contrast, the Reynolds number for the experimental apparatus was approximately 120, i.e., within the laminar flow regime. While it may be argued that the unanticipated fouling results might be a function of the reduced Reynolds value, the mechanisms proposed to date for fish scale-induced drag reduction are dependent on their ability to maintain laminar flow at large Reynolds numbers. Therefore, even at small Reynolds numbers, drag reduction-induced fouling resistance is expected to be present. These findings suggest that fishes might rely on alternate mechanisms for fouling resistance or the bioinspired asymmetric triangular riblets are incapable to perform the anticipated dual function of drag reduction and fouling resistance.

## 5. Summary and Conclusions

Novel bioinspired asymmetric triangular riblet surfaces have been shown to exhibit vortex drag reduction and a delayed transition to turbulence through numerical simulations. Optimal dimensions are largely dependent on the flow conditions and past studies proposed that drag reduction performance increases with Reynolds number with a maximum achieved performance of 9.1% compared to a flat surface. The holistic approach presented in this study includes all phases of research and development focused on fouling resistant microtopographies. This represents a departure from past attempts that were focused on limited aspects related to fouling resistant surfaces. The framework presented in this work can be used as a foundation for future modeling studies to provide designs capable to provide the desired functionality and manufacturability at micro/nano-scales.

To investigate the connection between drag reduction and fouling resistance, three samples were fabricated from PMMA. The results obtained suggest that the analyzed microstructures do not exhibit fouling resistant characteristics under the low Reynolds conditions yielded by the experimental setup used. Nonetheless, this study cannot be considered conclusive such that future experimental studies at higher Reynolds numbers will be conducted to further investigate the potential of the fabricated ATR samples. The following conclusions can be drawn from this study:

- Drag reduction was achieved for the full range of dimensions and Reynolds numbers considered in the numerical simulations
- While the experimental differences were rather small and therefore less conclusive, the trials performed suggested that the low Reynolds drag reduction is associated with smaller feature heights when compared to their higher Reynolds counterparts
- Drag reduction increases with the Reynolds number
- ATR structures can be fabricated through micromilling; their form accuracy was ±7 µm whereas the lowest areal roughness was *S_a_* = 85.2 nm
- The fouling resistance trials suggest that structures larger than the contaminating particles are more effective at reducing the overall settlement

Future work will focus on the development of an experimental apparatus that enables a higher channel velocity in order to be able to match the Reynolds numbers considered in the numerical simulations. This will allow the experimental verification of the drag reduction as well as the investigation of fouling resistance in the drag reduction region. Furthermore, the parametric study will be expanded to determine optimal feature dimensions with regard to drag reduction performance.

## Data Availability

Not applicable.
